# Cell patterning *in vivo* using microrobot specifically designed for tissue engineering applications

**DOI:** 10.1016/j.mtbio.2025.102683

**Published:** 2025-12-17

**Authors:** Hironori Yamazoe, Yoshiaki Yamano, Yuji Teramura, Shinichiro Shinzaki

**Affiliations:** a Molecular Biosystems Research Institute, National Institute of Advanced Industrial Science and Technology (AIST), 1-8-31 Midorigaoka, Ikeda, Osaka 563-8577, Japan; b Cellular and Molecular Biotechnology Research Institute, National Institute of Advanced Industrial Science and Technology (AIST), 1-1-1 Higashi, Tsukuba, Ibaraki 305-8566, Japan; c Department of Gastroenterology, Faculty of Medicine, Hyogo Medical University, 1-1 Mukogawa, Nishinomiya, Hyogo 663-8501, Japan

**Keywords:** Cell patterning, Tissue engineering, Stem cell therapy, Microrobot, Minimally invasive therapy, Protein-based material, Inflammatory bowel disease

## Abstract

The arrangement of cells in a desired pattern at target positions, known as cell patterning, is a crucial technique for constructing desirable tissues. While cell patterning has been traditionally performed on substrates, *in vivo* approaches remain largely unexplored. *In vivo* cell-patterning techniques show potential for achieving accurate and reliable tissue regeneration by building new tissues at lesion sites in a highly controlled manner using various therapeutic cells. This study introduces a pioneering approach for cell patterning *in vivo* using a microrobot specifically designed for tissue engineering applications. The body of the microrobot was fabricated using serum albumin and magnetic nanoparticles, and cell membrane-anchoring regents were bound to its surface. This robot effectively captured cells and rapidly released them at target sites, minimizing the burden on the recipient. Using this robot, cellular patterns formed successfully on various biological components, including Matrigel, other cell types, and inflamed colon tissues, in 30 min. Furthermore, as a proof-of-concept, cell patterning was performed inside the colon of mice with colitis. To ensure clinical applicability, the cell-loaded microrobot was introduced near the target site *via* an endoscope and subsequently guided by a magnetic field to create stem cell patterns in damaged colon tissues. This microrobot-based cell patterning will contribute to the establishment of a new field, *in vivo* cell patterning, and the advancement of sophisticated stem cell-based therapies.

## Graphical abstract

**Figure:**
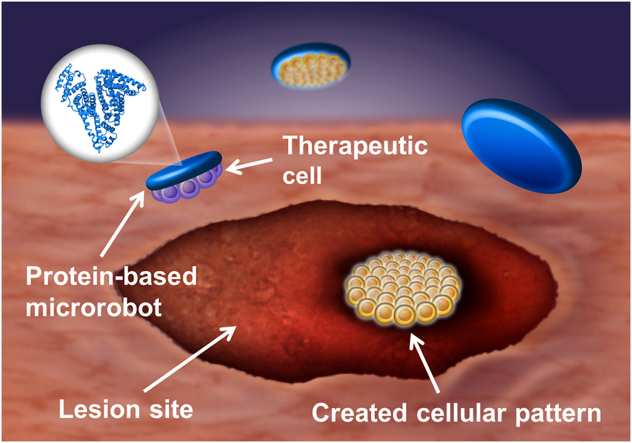

## 1 Introduction

The arrangement of cells according to a desired pattern at target positions, called cell patterning, is a key technique in the field of tissue engineering. This technique allows the fabrication of cell-based constructs utilized in various applications, including biomimetic artificial tissues that replicate the complex organization of *in vivo* tissue architectures for the replacement of damaged tissues [1], cell arrays and organs-on-chips for high-throughput screening of drug candidate libraries and cellular functions [[2], [3], [4]], and cellular assemblies for fundamental investigations of cell-cell communication [5]. These cell-based constructs are typically fabricated on substrates; therefore, methodologies for cell patterning have been developed to arrange various types of cells with arbitrary patterns on the substrates; these methods include inkjet printing [1,[6], [7], [8]], photolithography [6,9,10], microcontact printing [6,9,11], microfluidic patterning [6,9,12], microtopographies [6,9], patterned surfaces [6,9,13], dielectrophoresis [9], laser photoablation [14], surface acoustic wave [15], and optical tweezers [16]. Although cell patterning has been performed on substrates (i.e., outside the body), there are no practical methods for patterning cells inside the body.

Remarkable progress has been made in stem cell-based therapies over the past few decades. Transplantation of stem cells holds promise for regenerating extensive tissue damage in diverse organs, including the intestines, blood vessels, central nervous system, heart, and liver [[17], [18], [19], [20], [21]]. However, current strategies usually use only stem cells to repair damaged tissues, despite the fact that numerous cells, such as macrophages, neutrophils, fibroblasts, and epithelial cells, are involved in the tissue repair process [22,23]. Using this simple method, the tissue repair process cannot be precisely controlled, resulting in defective tissue regeneration. Tissue construction inside the body utilizing the cell-patterning technique, that is, the arrangement of various types of cells involved in tissue repair on arbitrary positions of the lesion sites with desired patterns, can reveal the roles of individual cells and interactions between these cells in the tissue repair process, as well as build new tissues in a highly controlled manner based on accumulated knowledge. *In vivo* cell-patterning techniques have great potential for realizing more accurate and reliable tissue regeneration than that achieved by existing stem cell therapies. Moreover, because cells are directly placed on lesion sites, the constructed tissues are well-fitted to a target tissue surface, unlike when tissue constructs prepared outside the body are implanted, in which an undesirable mismatch between the externally prepared tissue and target tissue surface is imperative [24].

Recently, we proposed a progressive approach to enable cell patterning not only on a substrate but also inside the body. We also developed an untethered mobile microrobot specifically designed for tissue engineering applications that can deliver cells to target positions with desired patterns beyond simple target delivery [25]. In previous studies, we successfully produced intricate cellular patterns composed of multiple cell types on substrates using a robot [25]. Building upon the successful *in vitro* results, in this study, we demonstrated cell patterning *in vivo* using our microrobot-based cell-patterning technique. Microrobots are often associated with untethered self-propelling mechanical robots capable of accessing target sites in the body and performing medical procedures—a perception influenced by the classic science fiction movies such as “Fantastic Voyage” and “Inner-space,” in which miniaturized submarines are injected into the human body to perform noninvasive surgery. However, the materials available for fabricating medical robots are limited because such materials must be nontoxic and nonimmunogenic [26,27]. Due to this limitation, many materials commonly used for the fabrication of robots, such as metals, silicone, and synthetic polymers, are unsuitable [28]. Precise manipulation of microrobots inside the body is technically challenging. Useful systems that allow remote navigation of tiny robots to target sites in deep regions of the body have not been established [29]. In addition, the motion of tiny robots is substantially affected by the highly viscous forces of fluids at low Reynolds numbers, which require a large amount of power for their movement [28,30,31].

To overcome these limitations and establish a practical method for cell patterning inside the body using a microrobot, we have created a highly biocompatible microrobot using a clinical-use grade protein. Additionally, we utilized an endoscope to introduce the microrobot near the target positions in the body to compensate for the difficulty of precise remote navigation of tiny robots over long distances. Medical devices, such as endoscopes and catheters, can access various target sites in the body through the blood vessels, gastrointestinal tract, and ureter in a minimally invasive manner. Therefore, the combination of these medical devices with microrobots is one of the worthwhile approaches to build clinically practicable microrobot-based medical systems [32,33].

As a proof of concept, we performed cell patterning inside the intestinal tract of an inflammatory bowel disease (IBD) mouse model. IBD, including ulcerative colitis and Crohn's disease, is a chronic relapsing inflammatory disorder of the intestinal tract that causes extensive damage to intestinal mucosal tissues [18,34]. We focused on IBD because recent studies showed that transplantation of stem cells such as intestinal stem cells and mesenchymal stem cells (MSCs) *via* intravenous, intraperitoneal, and rectal administration can promote the repair of damaged tissues, drawing increasing attention to stem cells for IBD treatment [18,[34], [35], [36]]. To the best of our knowledge, this is the first study to develop a practical method for patterning cells *in vivo*. This work will greatly contribute to the pioneering of a novel research area: *in vivo* cell patterning in the field of tissue engineering as well as the development of sophisticated stem cell-based therapies utilizing cell-patterning techniques.

## 2 Results and discussion

### 2.1 Microrobot design and cell patterning inside the intestinal tract using the microrobot

**Fig. 1:**
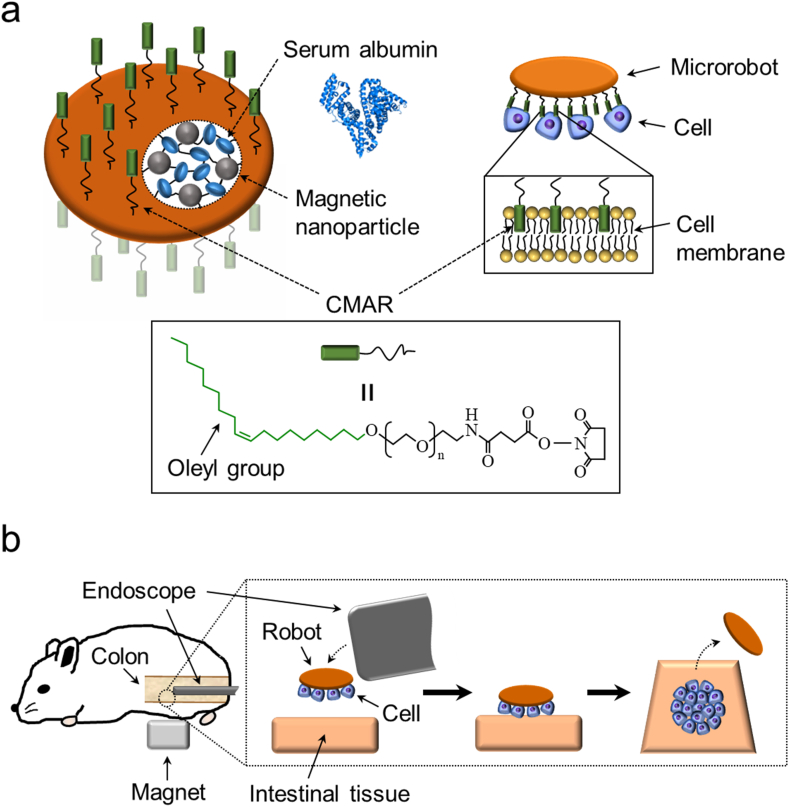
(a) Schematic illustration of the microrobot specifically designed for tissue engineering applications and cell capturing by the robot. (b) Process for pattering of cells inside the colon of the colitis mouse model using the microrobot and endoscope. Note that the cells are captured on both sides of the microrobot; however, only those on one side are used for patterning. The illustration presents the approach in an easily comprehensible manner.

A microrobot specifically designed for tissue engineering applications was fabricated using recombinant human serum albumin, a cell membrane-anchoring reagent (CMAR), and magnetic nanoparticles (MNPs) according to a method developed in our previous studies [25,37]. Fig. 1a shows a schematic diagram of the microrobot and capture of cells by the robot. The CMAR was immobilized on the surface of the microrobot for cell capture and release. The CMAR consists of a hydrophobic oleyl group, a polyethylene glycol spacer, and an amine-reactive group. The oleyl group in the CMAR interacts with the cell membrane through hydrophobic interactions, allowing the microrobot to capture cells. However, because the oleyl group–cell membrane interactions are weak and hydrophobic, cells captured by the microrobot can be released at the target site. The main body of the microrobot was fabricated using serum albumin for the following reasons. (1) Albumin has been used for approximately half a century to develop biocompatible drug delivery carriers, and some albumin-based carriers have been used in clinical settings [38,39]. (2) Clinical-use grade human albumin can be produced on a large scale using recombinant technology [40]. To create safe and reliable medical robots, recombinant proteins are superior to proteins obtained from natural sources because they can overcome limitations such as unstable supply, lot-to-lot variations, and contamination risks from prions, viruses, and mycoplasmas [41]. (3) Albumin possesses functional groups such as amino and carboxyl groups, which can be used to immobilize various molecules on the microrobot. The CMAR was covalently immobilized on the surface of our robot *via* amine-reactive groups in CMAR. (4) Albumin has the property of preventing cell adhesion on its coated surface [42,43]. Our albumin-based robot body was prepared while retaining the cell-nonadhesive property of native albumin. Therefore, the cells captured by the microrobot did not attach irreversibly to the robot, allowing the release of cells from the robot. MNPs, maghemite (γ-Fe_2_O_3_) particles with an average diameter of 50 nm, were incorporated into the robot body, conferring magnetic responsiveness to the microrobot. Maghemite nanoparticle are highly biocompatible and are clinically used as contrast agents in magnetic resonance imaging (MRI) [44,45].

Fig. 1b shows a schematic diagram of the process flow for cell patterning inside the mouse colon. A cell-capturing microrobot was introduced over the target region inside the colon of a mouse model of colitis using an endoscope. The microrobot was moved toward the luminal surface of the intestine and firmly attached to the intestinal tissue through the attractive force of a neodymium magnet placed outside the mouse. Because the microrobot simply moved toward the magnet over a short distance, complicated steering of the robot using a magnetic field was not necessary. By transferring cells from the microrobot to the intestinal tissue, cellular patterns with the same shape as those of the robot can be created on the intestinal surface.

### 2.2 Fabrication of the microrobot optimized for *in vivo* cell patterning

**Fig. 2:**
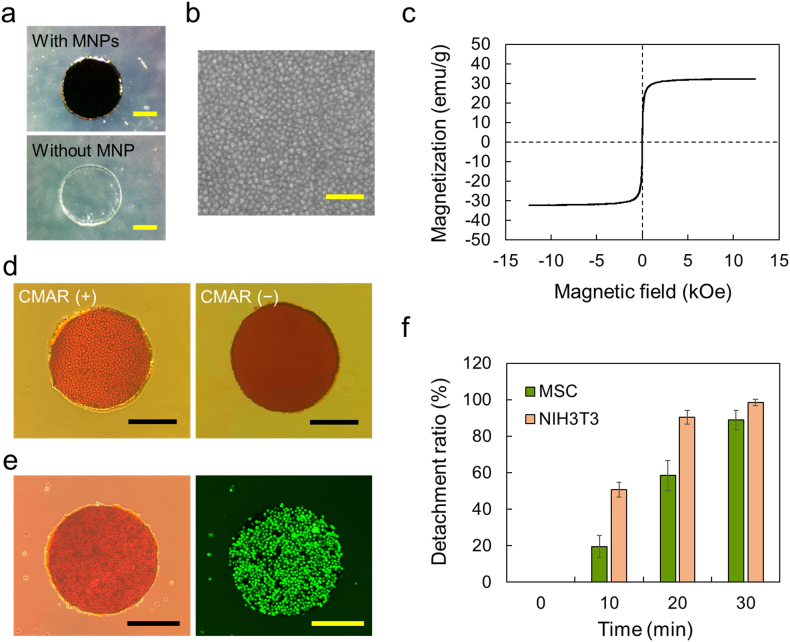
Magnetically responsive microrobot possessing cell capture-and-release property. (a) Images of robot bodies with and without MNPs. Circular-shaped bodies were prepared by cutting albumin films using the biopsy punch. Scale bar: 200 μ m. (b) SEM image of a surface of the microrobot body. Scale bar: 200 nm. (c) Magnetic hysteresis curve of robot body containing MNPs. (d) Phase-contrast micrographs of NIH3T3 cell-capturing microrobot and CMAR-devoid microrobot in which cells were not captured. Scale bar: 300 μ m. (e) Phase-contrast and fluorescence images of MSC-capturing microrobot. Scale bar: 300 μ m. (f) Detachment ratio of NIH3T3 cells and MSCs from robots after exposure to culture medium containing serum for 10, 20, and 30 min. Data are shown as the means ± SD (n = 6).

Our microrobot was fabricated using the following steps: (i) preparation of water-insoluble albumin films containing MNPs, (ii) cutting of the films to produce robot bodies with the desired shapes, and (iii) immobilization of CMAR on the surface of the robot bodies. In step (i), because films prepared from native albumin easily dissolve in water, cross-linked albumin was used to obtain water-insoluble films. Cross-linking of albumin was achieved using the method optimized in our previous studies, in which, to avoid excess cross-linking that leads to disruption of the structural and functional characteristics of native albumin, albumin was reacted with a cross-linker possessing epoxy groups at the lowest concentration required to prepare a water-insoluble film [43,46]. The resultant cross-linked albumin solution was mixed with the MNPs, cast onto a substrate, and dried overnight to form an MNP-containing film. Robot bodies with the desired shapes were then prepared by cutting the films using a biopsy punch and laser cutter. As shown in Fig. 2a and Fig. S1, robot bodies of various shapes can be easily produced by changing the film cutting design. Because MNPs absorb light, the robot body containing MNPs is dark brown in color, whereas the MNP-devoid body is transparent and colorless (Fig. 2a) [47]. Scanning electron microscopy (SEM) observation revealed a uniform distribution of bright spots, which was found to be consistent with the size of the incorporated MNPs (Fig. 2b). Furthermore, low-magnification energy-dispersive X-ray spectroscopy (EDS) mapping confirmed an even distribution of iron throughout the microrobot body, supporting the uniform incorporation of MNPs into the microrobot (Fig. S2).

The magnetic properties of the robot body were investigated using a vibrating-sample magnetometer. Fig. 2c shows the magnetic hysteresis curves of the robot body. No coercivity or remanence was observed in the magnetization curve, suggesting that the characteristic superparamagnetic behavior of the MNPs was maintained in the robot body. The saturation magnetization value was 32.24 emu/g, which was lower than that of pure MNPs (110 emu/g) because of the non-magnetic property of the albumin surrounding the MNPs. As illustrated in Fig. 1b, cell patterning was performed inside a mouse colon using a magnetically responsive robot. To achieve this, the microrobot inside the colon needed to be attracted to a magnet placed outside the mouse. The distance between the microrobot inside the colon and the magnet was approximately 7 mm. Therefore, we fabricated the microrobot to have strong magnetism by incorporating a much larger number of MNPs into the robot body compared to that in a robot in our previous studies, in which we created cellular patterns on the substrates using a robot with weak magnetism (saturation magnetization value, 0.67 emu/g), as it was placed in close proximity (approximately 0.9 mm) to the magnet underneath [25,37]. As shown in Movie S1, the circular microrobot was quickly attracted to the neodymium magnet placed 2.2 cm away, demonstrating the excellent magnetic responsiveness of the robot to the external magnetic field.

Additionally, when cell patterning is performed inside the bodies of living organisms, expediting the entire process is crucial to minimize the burden on the recipient. To accomplish cell patterning using a microrobot in a short time, the amount of CMAR immobilized on the surface of the robot is a key factor. When the amount of CMAR is low, the microrobot could not sufficiently capture cells in the initial cell-capturing step. By contrast, a large amount of CMAR prolonged the time required for the release of cells from the microrobot during the subsequent cell-releasing step. In our previous studies, an adequate amount of CMAR was immobilized to the robot surface without considering the reduction in the processing time, and the procedure was time-consuming (approximately 12 h) [25,37]. To determine the optimal amount of CMAR, a pilot study was conducted using robots prepared with various CMAR concentrations. The results indicated that microrobots with suitable cell capture-and-release properties could be prepared using 0.5 and 1 μ M CMAR for MSCs and NIH3T3 cells, respectively. These microrobots initially captured cells without any loss and released the captured cells after 30 min. Cell capture can be achieved by mixing the microrobot with a cell suspension. As shown in Fig. 2d, NIH3T3 cells were attached to the entire surface of the microrobot. This suggests that CMAR was successfully immobilized across the entire surface. The surface coverage of CMAR was examined using a fluorescent labeling dye (Fig. S3). No cell capture was observed in the control robot without CMAR immobilization, demonstrating that CMAR is required for cell capture. MSCs stably expressing green fluorescent protein (GFP) were also successfully captured by the microrobot, as observed in the phase-contrast and fluorescence images (Fig. 2e). The captured cells were detached from the microrobot by immersion in cell culture medium, as interaction between the cell membrane and oleyl group in CMAR was weakened due to the change of the interaction partner of the oleyl group from the cell membrane to the serum proteins [48,49]. Fig. 2f shows the detachment behavior of cells captured by the robot. Cell detachment was observed after 10 min of incubation at 37 °C, and almost all cells were detached after 30 min. Interestingly, the optimal amount of CMAR immobilized on the robot surface differed depending on the cell type. Quartz crystal microbalance (QCM) measurements showed that the amount of immobilized CMAR in the microrobot of NIH3T3 cells was 1.8-fold higher than that of MSCs (Fig. S4). By contrast, the results of image analysis showed that the area of a single cell captured by the microrobot was 2.2-fold larger in MSCs than in NIH3T3 cells (Fig. S5). As a result, a similar number of CMAR molecules (approximately 2 × 10^7^ CMAR molecules) was predicted to be incorporated into the cell membranes of both single cells. Although more detailed investigations are required, this value can serve as an indicator for obtaining microrobots that are well-suited for various types of cells.

### 2.3 Patterning of cells on various biological components using the microrobot

**Fig. 3:**
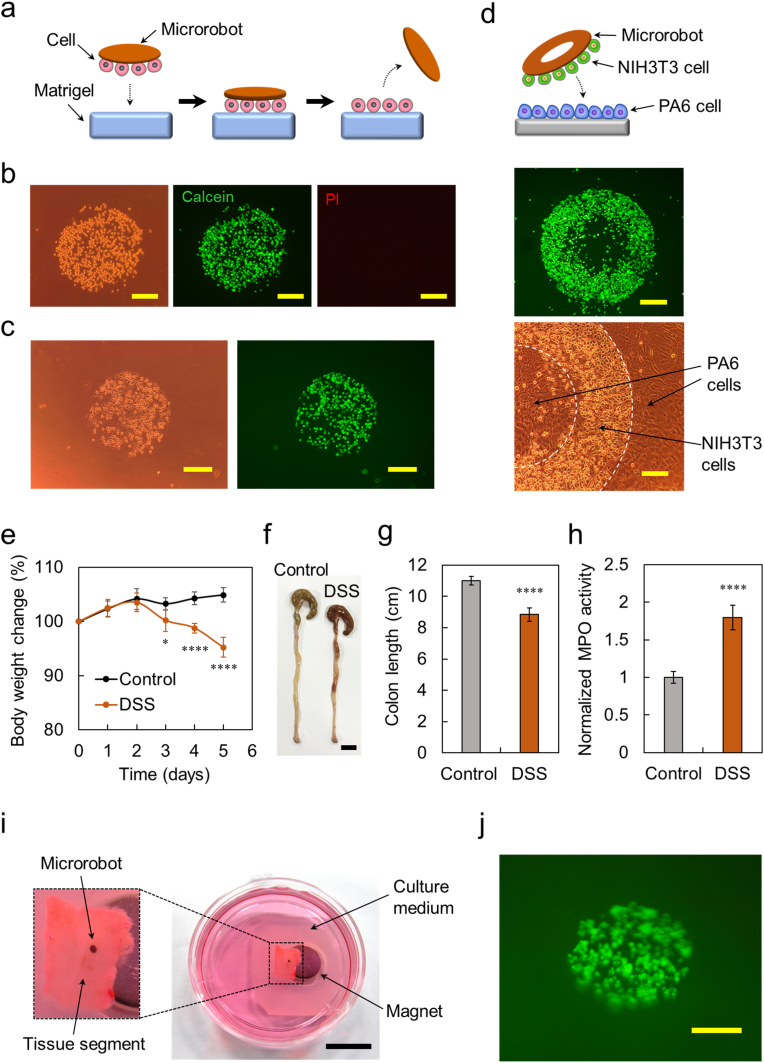
Creation of cellular patterns on various biological components. (a) Schematic illustration of the creation of cellular patterns on Matrigel using the microrobot. (b) Phase-contrast image of the circular pattern of NIH3T3 cells created on Matrigel and fluorescence images of patterned NIH3T3 cells stained with calcein-AM (green) and PI (red). Scale bar: 200 μ m. (c) Phase-contrast and fluorescence images of the circular pattern of MSCs created on Matrigel. Scale bar: 200 μ m. (d) Fluorescence image of the annular shaped pattern of NIH3T3 cells created on PA6 cells and its magnified phase-contrast view. Top panel scale bar: 300 μ m. Bottom panel scale bar: 100 μ m. (e–h) Induction of colitis in mice by DSS. Control: healthy control mice. DSS: DSS-treated mice. (e) Body weight changes after DSS induction of colitis. Mouse body weight was normalized as a percentage of day zero body weight. Data are presented as the means ± SD (n = 6 per group). ∗*p* < 0.05, ∗∗∗∗*p* < 0.0001 vs. control by two-sided Welch's *t*-test. (f) Macroscopic appearance of colons. Scale bar: 1 cm. (g) Length of colons on day 5. Data are presented as the means ± SD (n = 6 per group). ∗∗∗∗*p* < 0.0001 vs. control by two-sided Welch's *t*-test. (h) MPO levels in colon tissues on day 5. MPO activity is expressed as fold change relative to the control sample. Data are presented as the means ± SD (n = 6 per group). ∗∗∗∗*p* < 0.0001 vs. control by two-sided Welch's *t*-test. (i) Experimental setup for patterning of MSCs on inflamed colon tissues *ex vivo*. Scale bar: 1 cm. (j) Fluorescence image of the circular pattern of MSCs created on the inflamed colon tissues. Scale bar: 200 μ m. (For interpretation of the references to color in this figure legend, the reader is referred to the Web version of this article.)

Using our microrobot, cells can be arranged at target positions with desired patterns in a short time through their attachment to the target positions and subsequent detachment from the robot. Generally, the cells adhere stably to a substrate for several hours while forming focal contacts. In order to achieve rapid attachment of the microrobot-captured cells to the target surfaces, we utilized physical adsorption instead of a time-consuming focal-contact-mediated adhesion process. In physical adsorption, cells attach to surfaces *via* physical interactions, such as electrostatic, hydrogen bonding, hydrophobic, and van der Waals forces interactions, that are instantaneously formed as the cells and target surfaces come into close proximity. We initially performed cell patterning on Matrigel using microrobots. Matrigel is a protein mixture of matrix components from mouse sarcomas and is commonly used in *in vitro* models of biological tissues [50]. As illustrated in Fig. 3a, the cell-loaded microrobot was placed on Matrigel, and the cells captured by the robot were in tight contact with the gel surface by the attraction force of the magnet placed under the gel, which promoted physical adsorption of the cells to the gel. The cells were then exposed to culture medium containing serum for 30 min, which induced detachment of cells from the robot. Consequently, the cells captured by the microrobot were transferred onto the gel surface, producing a cellular pattern of the same shape as that of the robot. As shown in Fig. 3b and c, circular patterns of NIH3T3 cells and MSCs were successfully created on the gels using circular microrobots in 30 min. The series of processes used during cell patterning did not cause damage to NIH3T3 cells, as indicated by the intense staining of patterned cells with calcein-acetoxymethyl ester (calcein-AM), but not propidium iodide (PI) (Fig. 3b). The percentage of the cell coverage of NIH3T3 cells within the predetermined circular pattern was 83 ± 3 %. Green fluorescence derived from GFP was observed in the cellular pattern of MSCs expressing this protein (Fig. 3c). The coverage of the MSCs in the circular pattern was 67 ± 4 %. We further performed cell patterning on different cell types (Fig. 3d). In this experiment, a PA6 cell layer without any gaps was prepared by culturing PA6 cells until confluent to eliminate the possibility that NIH3T3 cells placed on PA6 cells were partly attached to the underlying substrate but not PA6 cells. NIH3T3 cells, previously stained with a green fluorescent dye, were delivered onto the PA6 cell layer using an annular-shaped robot. An annular-shaped pattern of NIH3T3 cells was created on PA6 cells in 30 min through transfer of the delivered NIH3T3 cells to the PA6 cell layer. Attachment of round-shaped NIH3T3 cells to elongated PA6 cells was also clearly observed in the magnified view of the phase-contrast image.

Subsequently, we performed cell patterning of the inflamed colon tissue *ex vivo* using a mouse model of dextran sulfate sodium (DSS)-induced ulcerative colitis. This model is widely used because it exhibits numerous phenotypic features relevant to human ulcerative colitis [51]. To induce colitis, mice were administered DSS in drinking water for 5 days. DSS-treated mice showed typical signs of colitis development, such as body weight loss, diarrhea, and bloody stools. As shown in Fig. 3e, a decline in body weight was observed in DSS-treated mice from day 3 of DSS administration until the end of the experimental period. In contrast, weight increased continuously in healthy control mice. In this model, the degree of inflammation was indicated by shortening of the colon length. Colon length is a widely recognized indicator of the progression and severity of colitis [[52], [53], [54], [55]]. After 5 days of DSS administration, the colon length in DSS-treated mice was shorter than that in the control group mice (Fig. 3f and g). Myeloperoxidase (MPO) is an enzyme present in neutrophils, and its activity within the colon reflects the level of neutrophil infiltration. Therefore, the degree of colon inflammation was quantitatively determined by measuring MPO activity [[52], [53], [54], [55]]. As shown in Fig. 3h, MPO activity in DSS-treated mice was 1.8-fold higher than that in the control mice. These findings are consistent with earlier studies of DSS-induced colitis in mice, indicating the successful induction of colitis using DSS [[52], [53], [54], [55]]. Fig. 3i shows the experimental setup for the cell patterning of inflamed colon tissues *ex vivo* using a microrobot. Inflamed colons were excised from the prepared colitis mice, and their distal portions were cut into small segments. The tissue segment was opened longitudinally and placed in a dish with the luminal side facing upward. A neodymium magnet was then set under the dish. An MSC-loaded circular microrobot was placed on the tissue segment and firmly contacted the tissue surface by a magnetic attraction force, followed by exposure to culture medium containing serum for 30 min. As shown in Fig. 3j, the cellular pattern of MSCs was successfully created on the inflamed colon tissue, forming a clear and distinct circular arrangement on the tissue surface.

Thus, cellular patterns can be created on various biological components, including Matrigel, other cell types, and inflamed colon tissues using microrobots in 30 min. Notably, because the cells were attached to the target sites *via* physical adsorption, their efficiency was highly dependent on the physicochemical properties of the target surface. In fact, more precise cellular patterns were produced on Matrigel than on collagen gel, possibly because of differences in the charge, concentration, and matrix-binding sites for cellular receptors of gel matrix components (Fig. S6). Inflamed colon tissue is rich in positive charge because of the accumulation of positively charged molecules, such as transferrin, bactericidal/permeability-increasing proteins, and antimicrobial peptides, which can facilitate cell attachment to tissues *via* electrostatic interactions [[56], [57], [58]]. A useful approach to improve attachment efficiency is to modify the surface properties of the cells for patterning. As shown in Fig. S7, the amount of positive charge can be increased by immobilizing positively charged peptides on the cell surface to promote the attachment of cells to the polystyrene culture dish, which has a negatively charged surface, resulting in the creation of a precise cellular pattern.

### 2.4 Patterning of stem cells *in vivo* using the microrobot

**Fig. 4:**
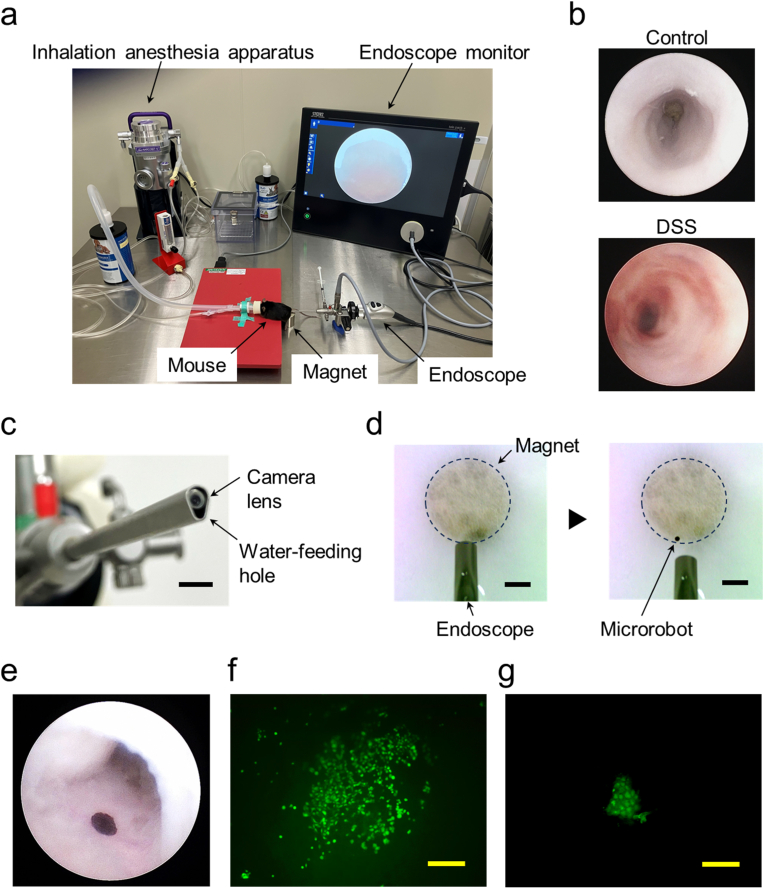
Patterning of stem cells inside the mouse colon. (a) Experimental setup for patterning of MSCs inside the colon of colitis mouse using the microrobot and endoscope. (b) Endoscopic images of colons of a healthy control mouse and DSS-treated mouse. (c) Magnified view of the tip of the endoscope. Scale bar: 3 mm. (d) Time-lapse images of release of the circular microrobot with 500 μ m-diameter from the endoscope. Thin white paper was placed on the magnet so that the microrobot with dark brown color can be clearly observed. Scale bar: 3 mm. (e) Endoscopic image of MSC-loaded circular microrobot with 500 μ m-diameter placed inside the colon. (f) Fluorescence image of the circular pattern of MSCs created on the inflamed colon tissue. Scale bar: 200 μ m. (g) Fluorescence image of pattered MSCs 9 days after patterning of the cells on the inflamed colon tissue. Scale bar: 200 μ m. (For interpretation of the references to color in this figure legend, the reader is referred to the Web version of this article.)

Finally, we performed cell patterning inside the colons of DSS-induced colitis mice using a microrobot and an endoscope, as illustrated in Fig. 1b. The experimental setup is shown in Fig. 4a. Mice with colitis were anesthetized using an inhalation anesthesia apparatus, and an endoscope was inserted into the rectum. Endoscopic observation revealed inflammation in the colons of colitis mice but not in the healthy control mice (Fig. 4b–Movie S2, Movie S3). Fig. 4c shows the tip of the endoscope equipped with a camera lens and water-feeding hole. The cell-loaded microrobot, suspended in phosphate-buffered saline (PBS), was introduced over the target region inside the colon from the water-feeding hole of the endoscope. The time-lapse images in Fig. 4d, corresponding to Movie S4, show that the microrobot released from the endoscope was attracted to the magnet and attached to a position just under the release point. As shown in Fig. 4e and Movie S5, the MSC-loaded circular microrobot was introduced into the colon and firmly attached to the surface of the inflamed colon *via* the attractive force of a neodymium magnet placed outside the mouse. Cells captured by the robot were then transferred to the colon tissue by exposure to culture medium containing serum for 30 min. Our protein-based microrobot was not designed for rapid degradation *in vivo*, primarily in order to ensure reliable cell patterning and mitigate the risk of releasing encapsulated MNPs. The stability of the microrobots was confirmed *in vitro*; they did not degrade even after exposure to a high concentration of protease (Fig. S8). Additionally, as the main role of the colon is water absorption, protein breakdown within the colon is minimal. Therefore, after cell patterning, the microrobot was not retrieved and was simply excreted from the body along with the excreta. However, if needed, the microrobots inside the colon could be retrieved using endoscopic forceps with a magnet attached to the tip (Fig. S9). Movie S6 shows the retrieval capability of the magnetic forceps *in vitro*.

To evaluate the creation of cellular patterns, colons were excised from the mice and observed internally using a fluorescence microscope. As shown in Fig. 4f, a circular cellular pattern of MSCs was successfully created on the inflamed colon tissue. Furthermore, although the cell population was reduced, patterned MSCs persisted and were still detectable 9 days after cell patterning, demonstrating sustained cell survival (Fig. 4g). Effective cell engraftment and growth of stem cells, including MSCs and intestinal stem cells, on the inflamed colonic surface of colitis mice have been reported previously [36,59,60]. However, cells cultured *in vitro* are exposed to vastly different environments following cell patterning *in vivo*. Covering the created cellular patterns with clinically used polyglycolic acid-based bioabsorbable scaffold sheets and fibrin sealants may be useful to alleviate dramatic environmental changes and support the initial survival and subsequent proliferation of the patterned cells [61]. This covering material also protected the patterned cells from physical stress (Fig. S10). Although future studies, including quantitative evaluation of the long-term behavior of patterned cells, construction of complex tissues composed of multiple types of cells, and investigation of the effect of constructed tissues on the repair of damaged tissues, are required, we successfully demonstrated the patterning of stem cells inside the colon using a microrobot.

Using our approach, various cell types can be arranged in arbitrary patterns at lesion sites. In the intestine, telocytes reside beneath the epithelial layer and make contact with adjacent epithelial cells, thereby playing key roles in regard to intestinal development, stem cell niche maintenance, and intestinal tissue repair [23]. Therefore, a multilayered cellular assembly that mimics the *in vivo* environment prepared by patterning telocytes and subsequently placing intestinal stem cells in the established pattern represents a promising design strategy. Previous studies have indicated that combined transplantation of MSCs and hematopoietic stem cells (HSCs) facilitates regeneration of the intestinal epithelium [62]. The co-culture of MSCs with intestinal epithelial cells promotes their differentiation into intestinal epithelioid cells [63]. Therefore, the preparation of cellular patterns composed of patterned MSCs surrounded by partnering cells such as intestinal epithelial cells or HSCs is another meaningful design strategy. In addition, in our method, the cells captured by the microrobot are directly placed on the target tissues and cells, thus allowing cell-to-tissue and cell-to-cell contact. Because these direct contacts are essential for mutual interactions, our method is advantageous compared to placing cells embedded in scaffolds such as hydrogels on target objects, as these scaffolds interfere with this contact [24,64,65]. It has been reported previously that highly efficient stem cell differentiation toward specific lineages and enhanced cellular function were facilitated *via* direct cell-to-cell contact [66,67]. Therefore, we expect that our cell-patterning approach has great potential to yield superior therapeutic efficacy compared to conventional scaffold-based stem cell transplantation.

However, unlike cell transplantations using scaffolds, patterned cells are directly exposed to harsh stimuli within the intestinal lumen and lack adequate structural support for their growth. This likely contributed to the low survival rate of the patterned cells (Fig. 4g). We have hypothesized that these limitations could be overcome by covering the patterned cells with scaffold materials, as aforementioned. Magnetically actuated microrobots serving as scaffolds have been developed to deliver therapeutic cells to target sites in the body while simultaneously providing a suitable environment for their growth [47,68]. However, a major concern regarding these scaffold-based microrobots is their potential long-term retention in the body, which may induce adverse immune responses. By contrast, our approach, in which the microrobot is solely dedicated to cell placement and does not serve as a long-term scaffold, offers a significant advantage in mitigating this risk. Furthermore, the use of autologous cells for cell patterning minimizes the risk of adverse immune responses from repeated administration because it avoids issues related to immunogenicity. Autologous stem cell transplantation has been performed in ulcerative colitis patients [34,69].

In this study, we mainly used circular microrobots with 500-μ m diameter. Because microrobots of this size were prepared by cutting large freestanding films, larger robots can be easily fabricated. Although tiny microrobots (with sizes below 400 μ m) are difficult to prepare by cutting films, they can be fabricated by depositing small droplets of cross-linked albumin solution containing the MNPs on the substrate using inkjet printing [25,37]. In our previous studies, we successfully fabricated circular microrobots with 50-μ m diameter using this technique. This size was deemed sufficiently small, considering that a typical single cell is approximately 10–30 μ m in size.

The accurate delivery and placement of cell-loaded microrobots to target sites within the human body is a critical challenge. The position of the endoscope tip must be precisely controlled because the microrobots are released from the tip of the endoscope. Given the inherent limitations of manual operations for precise positioning, robotic technology would be useful for achieving greater accuracy. In the colon, dynamic movements, such as peristalsis and segmentation, interfere with the accurate placement of microrobots at target sites. Therefore, prior to cell patterning, these movements should be suppressed using specific drugs, such as anticholinergics and glucagon.

The primary challenge in regard to human applications, such as targeting the intestines, lies in the significantly greater distance between the external magnetic source and microrobots compared with a mouse model. In order to overcome the drastically reduced magnetic field strength and gradient at the target site, two key enhancements are necessary. First, the magnetic responsiveness of the microrobots must be increased; and second, the simple permanent magnet must be replaced by a powerful external magnetic field generation system capable of providing the strong, focused fields and high gradients essential for manipulation deep within the human torso, such as a large electromagnet array as well as conventional clinical magnetically guided ablation catheter and MRI systems [[70], [71], [72], [73]]. However, because our approach requires the microrobot released from the endoscope to perform only short-distance, unidirectional movement toward the target tissue surface, the guidance system is simpler than systems that require complex three-dimensional or multi-degree-of-freedom rotational maneuvers, thereby providing a significant advantage in terms of practical feasibility.

## 3 Conclusion

We present a pioneering approach for patterning cells *in vivo* using a microrobot specifically designed for tissue engineering applications. For cell patterning within living organisms, a short procedural time is crucial to minimize the burden on the recipient. To achieve this, we fabricated a microrobot that initially captured cells and subsequently released them at target sites within a short time. Using this robot, cellular patterns were created on various biological components, including Matrigel, other types of cells, and inflamed colon tissues, in 30 min. Furthermore, we performed cell patterning inside the colons of mice with colitis using a microrobot and endoscope, successfully creating patterns of stem cells on damaged colon tissues. Considering the feasibility in a clinical setting, a microrobot with high biocompatibility was prepared using a clinical-use grade protein. Additionally, the cell-loaded microrobot was delivered to the target site in the colon without full remote navigation by introducing it near the target site *via* an endoscope and then guiding it with a magnetic field. Thus, the microrobot-based cell patterning presented in this study opens a new research area: *in vivo* cell patterning in the field of tissue engineering. *In vivo* cell-patterning techniques enable the construction of new tissues at lesion sites in a highly controlled manner using various types of cells involved in tissue repair, which has great potential for realizing more accurate and reliable tissue regeneration than that achieved by simple stem cell grafting. Safe, efficient, and minimally invasive therapy using therapeutic cells and minimally invasive medical tools such as miniature medical robots, endoscopes, and catheters is an ideal form of next-generation medical care.

## 4 Materials and methods

### 4.1 Preparation of the main body of microrobot using serum albumin and MNPs

A cross-linked albumin solution was prepared according to the method developed in our previous study with slight modifications [25]. Briefly, recombinant human serum albumin (Recombumin® Elite, Product code: 205-005; Albumedix Ltd., Nottingham, UK) was dissolved in PBS (pH 7.4) to yield a 30 mg/mL solution, which was then reacted with 215 mM ethylene glycol diglycidyl ether (EGDE; Wako, Osaka, Japan) with vigorous stirring for 24 h at 25 °C. The reaction mixture was dialyzed for 3 days at room temperature against Milli-Q water in cellulose tubing (molecular weight cutoff = 12 kDa; Nihon Medical Science, Gunma, Japan) to remove unreacted EGDE. The resultant cross-linked albumin solution was adjusted to a final concentration of 20 mg/mL by adding Milli-Q water, sterilized by filtering through a 0.22 μm filter, and stored at 4 °C. Amine-functionalized dextran-coated maghemite (γ-Fe_2_O_3_) MNP solution (synomag®-D, Product code: 104-01-501, average size = 50 nm; Micromod Partikeltechnologie GmbH, Rostock, Germany) (10 mg/mL, 60 μ L) was centrifuged at 17,400×*g* for 15 min. After removing the supernatant, the MNPs were resuspended in 20 μ L of the cross-linked albumin solution containing 0.95 % glycerol. This mixture was placed on silicon rubber sheets (AS ONE, Osaka, Japan) and dried overnight in a thermohygrostat (SH-222; ESPEC Co., Ltd., Osaka, Japan) at 37 °C and 50 % relative humidity to form water-insoluble albumin films containing MNPs. Freestanding films were obtained by peeling the films away from the silicon sheets. The films were cut using a biopsy punch (Kai Industries Co., Gifu, Japan) or laser cutter (Etcher Laser Pro; SmartDIYs Co., Ltd., Yamanashi, Japan) to obtain robot bodies with specific shapes. The prepared robot bodies swelled slightly when they were immersed in the aqueous solution. The magnetic properties of the robot bodies were measured using a vibrating-sample magnetometer (VSM-5; TOEI Industry Co. Ltd., Tokyo, Japan) at room temperature. SEM and EDS analyses were performed using a scanning electron microscope (Regulus 8220; HITACHI, Tokyo, Japan).

### 4.2 Immobilization of CMAR to the surface of robot bodies

The robot bodies were immersed in 0.5 or 1 μ M CMAR (SUNBRIGHT OE-040CS; *M*n = 4000, NOF Co., Tokyo, Japan) solution in PBS for 30 min at 25 °C to react the amine-reactive group in CMAR with the ε-amines of the lysine side chains and α-amines at the N-terminus of albumin molecules on the surface of robot body. The prepared robots were washed with PBS three times and stored at 4 °C. To evaluate the biodegradability of the microrobots, robots were incubated with 1 mg/mL porcine pancreas trypsin (1170 BAEE U/mg; Sigma, St. Louis, MO, USA) in PBS for 24 h at 37 °C. Binding of CMAR to the surface of the albumin-based robot bodies was investigated using a QCM equipped with an AT-cut QCM sensor with a gold surface (Single-Q; AS ONE). The sensor surfaces were cleaned with piranha solution, and a cross-linked albumin film was formed on the sensor surface. After equilibrating the sensor by exposure to PBS, 0.5 or 1 μ M CMAR in PBS was introduced into the measuring chamber. The change in oscillation frequency of the quartz crystal oscillator was monitored for 30 min. The temperature was kept at 25 ± 0.05 °C throughout the measurement. An increase in the mass attached to the QCM sensor surface decreased the oscillation frequency of the quartz crystal oscillator. The mass attached to the sensor surface at 30 min after adding CMAR was estimated using the following Sauerbrey equation [74]:

$$\Delta f=-\frac{2{f_{0}}^{2}}{A\sqrt{\rho_{q}\mu_{q}}}\Delta m\text{,}$$

where Δ*f* is the measured frequency change (Hz), *f*_*o*_ is the fundamental frequency of the quartz crystal oscillator (27 × 10^6^ Hz), Δ*m* is the mass change (g), *A* is the sensing area (4.9 mm^2^), ρ*_q_* is the density of quartz (2.65 g/cm^3^), and μ_*q*_ is the shear modulus of quartz (2.95 × 10^11^ dyn/cm^2^). The QCM data from 5 samples were averaged. In order to examine the surface coverage of CMAR, the microrobots for NIH3T3 cells before and after CMAR immobilization were treated with an amine-reactive fluorescent labeling dye using the AnaTagTM HiLyte FluorTM 488 protein labeling kit (AnaSpec, Inc. Fremont, CA, USA) according to manufacturer's instructions.

### 4.3 Cells and cell culture

Mouse NIH3T3 fibroblast cells (RCB1862; Riken Cell Bank, Tsukuba, Japan) were grown on culture dishes in Dulbecco's Modified Eagle's Medium (DMEM) supplemented with 10 % fetal bovine serum (FBS), 100 U/mL penicillin, and 100 μg/mL streptomycin. Mouse stromal PA6 cells (RCB1127; Riken Cell Bank) were grown on culture dishes in Alpha Minimal Essential Medium supplemented with 10 % FBS, 100 U/mL penicillin, and 100 μg/mL streptomycin. Green fluorescent protein-expressing C57BL/6N mouse bone marrow MSCs were purchased from Creative BioArray (Catalog no., CSC-C1314; Shirley, NY, USA). The cells were grown on culture dishes in SuperCult® mouse MSC growth medium (Creative Bioarray). All cells were cultured in a humidified 5 % CO_2_ incubator at 37 °C. The cells were observed using a phase-contrast microscope (IX70; OLYMPUS, Tokyo, Japan).

### 4.4 Capture of cells by the microrobot

Cultivated NIH3T3 cells or MSCs were collected from the dishes and resuspended in serum-free DMEM. To capture cells using the microrobot, one robot was mixed with 3 × 10^5^ cells in a small chamber, followed by incubation for 40 min, with the chamber turned upside down every 10 min. We used serum-free medium in the cell-capturing process because proteins in the serum prevent the interaction between the robot and cells, causing a decrease in the capturing efficiency. After removing the supernatant containing uncaptured cells, the cell-capturing microrobot was suspended in PBS. CMAR-devoid microrobots, prepared by omitting the binding of CMAR to robot bodies, were used as controls in the cell capture assay. The area of a single cell captured by the microrobot was determined by analyzing the microscopic images using ImageJ software (version 1.54 g; NIH, Bethesda, MD, USA). The cell area data from 30 samples were averaged.

### 4.5 Detachment behavior of cells from the robot

The cells captured by the robot could be detached from the robot after exposure to serum-containing culture medium. For quantitative analyses of the detachment of NIH3T3 cells and MSCs from the robots, CMAR-carrying albumin films were prepared on the plates using the same procedure as that for the preparation of the robot. The cross-linked albumin solution was poured onto a multiwell plate (0.95 cm^2^/well; Thermo Fisher Scientific, Waltham, MA, USA) and dried overnight to form an albumin film on the plate, followed by binding of CMAR to the film surfaces. NIH3T3 cells and MSCs suspended in serum-free medium were seeded into plates at a density of 5 × 10^4^ cells/cm^2^ and incubated for 10 min at room temperature to immobilize the cells on the CMAR-carrying surfaces. After washing with PBS, the serum-free medium was removed and replaced with DMEM containing 10 % FBS. The cells were then incubated at 37 °C. At 10, 20, and 30 min, the supernatants containing the detached cells were removed from the plates and fresh medium was added. Immobilized cells were counted using a phase-contrast microscope at each time point. The detachment ratio was estimated by dividing the number of immobilized cells after culturing for a predetermined period by the number of immobilized cells.

### 4.6 Cell patterning on the Matrigel and different types of cells using the microrobot

To perform cell patterning on Matrigel (Catalog no., 356234; Corning, NY, USA), Matrigel solution was added to the dishes and incubated in a CO_2_ incubator at 37 °C for 30 min for gelation. Next, NIH3T3 cells or MSC-loaded circular microrobots with a 500 μ m-diameter were placed on the gels and tightly contacted the gel surface using a neodymium magnet sheet (thickness, 0.4 mm; magnetic induction, 60 mT; Magna Co., Ltd., Tokyo, Japan) placed under the dishes. A weak magnetic sheet was used to prevent the microrobot from being pulled into the fragile gel. The microrobots placed on the gels were incubated for 30 min in DMEM containing 10 % FBS to transfer the cells onto the gel surfaces. After removing the microrobots without loading the cells from the dishes using a pipette, the created cellular patterns were observed using an IX70 phase-contrast and fluorescence microscope. The viability of the NIH3T3 cells after cell patterning was evaluated by staining the cells with calcein-AM (Dojindo Laboratories, Kumamoto, Japan) and PI (Dojindo Laboratories) according to the manufacturer's instructions. For cell patterning on collagen gels, gels were prepared using 2.4 mg/mL porcine type I collagen solution according to the manufacturer's instructions (Collagen gel culturing kit; Nitta Gelatin, Inc., Osaka, Japan). To assess patterning fidelity, we calculated the cellular coverage within the predetermined circular pattern by analyzing the microscopic images using ImageJ software (version 1.54 g).

To pattern NIH3T3 cells on PA6 cells, PA6 cells were cultured until confluent, and a NIH3T3 cells-loaded annular shaped robot (outer diameter, 1 mm; inner diameter, 0.5 mm) was placed on the PA6 cell sheet, followed by tight contact with the PA6 cells using a cylindrical neodymium magnet (diameter, 9 mm; height, 2 mm; magnetic induction, 243 mT; NeoMag Co., Ltd., Ichikawa, Japan). The NIH3T3 cells were fluorescently visualized using a cytoplasmic tracer (CFSE-green; Invitrogen, Carlsbad, CA, USA) prior to cell capture by the microrobot. NIH3T3 cells were transferred onto the PA6 cell sheet and the created cellular patterns were observed as described above.

### 4.7 Cell patterning on the inflamed colon tissue *ex vivo* using the microrobot

Six-week-old male C57BL/6N mice were purchased from CLEA Japan, Inc. (Tokyo, Japan). The mice were fed a standard laboratory diet (MF LID6; Oriental Yeast Co., Ltd., Tokyo, Japan) and tap water *ad libitum*. They were housed in a standard 12 h light/dark cycle at a room temperature of 23 ± 2 °C and relative humidity of 55 ± 15 %. The animals were acclimated to the laboratory environment for at least 1 week before the experiment. All experimental procedures were approved by the AIST Ethical Committee for Animal Experiments (no. 2025-0295).

Mice were fed 2.5 % DSS (MW 36,000–50,000; MP Biomedicals, Santa Ana, CA, USA) dissolved in drinking water *ad libitum* for 5 days to induce colitis. Fresh DSS solution was prepared in the morning. Control mice received normal drinking water. Mice were randomly assigned to the control and treatment groups. All mice were sex- and age-matched within each group. The mice were observed daily to monitor changes in body weight, as well as the appearance of diarrhea and bloody stools. Daily weight changes were calculated as percentages of initial weight. The mice were sacrificed after 5 days of DSS treatment. The inflamed colons were excised from the mice and flushed with PBS to clear the intestinal contents, and the colon lengths were measured.

To perform cell patterning on the inflamed colon tissues, the distal portion of the excised colon was cut into small segments. The tissue segment was opened longitudinally and placed in a dish with the luminal side facing upward. MSC-loaded circular microrobots with a diameter of 500 μ m were placed on the tissue segment and tightly contacted the surface of the tissue using a cylindrical neodymium magnet (diameter, 9 mm; height, 2 mm; magnetic induction, 243 mT; NeoMag Co., Ltd.) placed under the dish. MSCs were transferred onto the tissue surface by exposing the cell-loaded microrobot to DMEM containing 10 % FBS for 30 min. After removing the robots from the dishes without loading the cells using a pipette, cellular patterns were observed using an IX70 fluorescence microscope.

### 4.8 Measurement of MPO activity in inflamed colon tissue

Colon tissues obtained from healthy control and DSS-treated mice were homogenized using a homogenizer (Micro Smash MS-100; TOMY, Tokyo, Japan). The homogenates were centrifuged at 20,000×*g* for 15 min at 4 °C, and the supernatants were collected. MPO activity and protein concentration in the supernatants were measured using an MPO peroxidation activity assay kit (Biovision, Milpitas, CA, USA) and a Coomassie Brilliant Blue-based Protein Quantification Kit (Dojindo Laboratories), respectively, according to the manufacturer's instructions. MPO activity was normalized per milligram of protein and expressed as fold change relative to the control sample.

### 4.9 Cell patterning on inflamed colon tissue *in vivo* using the microrobot

Mice treated with DSS for 5 days were anesthetized with inhalation of isoflurane (induced at 3 % and maintained on 1 % isoflurane) and placed on a heating pad to maintain homeostasis. The colon was washed with 200 μ L of warm PBS using an oral gavage needle. We used a Coloview endoscope system (Karl Storz, Tuttlingen, Germany), consisting of a miniature endoscope (outer diameter, 1.9 mm) covered with an operating sheath, a triple chip camera, a xenon light source, and a color monitor, for the observation of the colon as well as for the introduction of the cell-loaded microrobot inside the colon. The endoscope was carefully inserted into the rectum while the endoscopic procedure was viewed on a color monitor. The MSC-loaded circular microrobot with a diameter of 500 μ m, suspended in PBS, was released over the target region inside the colon from the water-feeding hole of the endoscope, which was attracted to a cylindrical neodymium magnet (diameter, 30 mm; height, 30 mm; magnetic induction, 613 mT; NeoMag Co., Ltd.) placed outside the mouse. DMEM containing 10 % FBS was instilled into the colonic lumen of the mice to induce the transfer of cells from the microrobot to the colon tissues. Excretion of the microrobot without loading the cells outside the body was confirmed by collecting the excreta and mixing it with water, followed by separation of the robot from the mixture using a magnet. The treated mice were maintained under normal housing conditions with *ad libitum* access to food and water without DSS. No immunosuppressive agents were administered to recipient mice because syngenetic mice and MSCs were used in the experiment. Thirty minutes or nine days after cell patterning, the colons were excised from the mice. After flushing with PBS to clear the intestinal contents, the colons were opened longitudinally and sandwiched between glass slides for maximal flattening. This was performed to facilitate focusing during microscopic observations, although the inherent tendency of the tissue to revert to its original tubular shape and the uneven topography prevented a perfectly flat plane. Patterned cells in the colon tissues were observed using an IX70 fluorescence microscope.

### 4.10 *In vitro* physical stress assay

MSCs were seeded onto multiwell plates (0.95 cm^2^/well) at a density of 2 × 10^5^ cells/cm^2^. Two sample groups were prepared: one in which the cells were covered with 2.4 mg/mL porcine type I collagen solution and the other in which the cells remained uncovered. After the addition of 15 zirconia beads (2 mm in diameter; TOMY, Tokyo, Japan) to each well, the plate was laterally shaken at 200 rpm for 1 h in a rotary shaker (RS-2; AS ONE). Viable cells were then quantified using a cell counting kit-8 (Dojindo Laboratories) according to the manufacturer's instructions. The cell survival rate was calculated by dividing the viable cells in the shaken sample by those in the non-shaken control, independent of the covered or uncovered groups.

### 4.11 Statistical analysis

Quantitative values are expressed as the mean ± standard deviation. Statistical differences were identified by a two-sided Welch's *t*-test using Microsoft Excel (version 2506; Microsoft, Redmond, WA, USA). Differences were considered significant at *p v*alues < 0.05. All qualitative experiments such as cell capturing by the robot and cell patterning on various target objects were repeated independently at least three times with triplicate samples to ensure reproducibility.

## CRediT authorship contribution statement

**Hironori Yamazoe:** Writing – review & editing, Writing – original draft, Visualization, Validation, Supervision, Project administration, Methodology, Investigation, Funding acquisition, Formal analysis, Data curation, Conceptualization. **Yoshiaki Yamano:** Writing – review & editing, Visualization, Validation, Investigation, Formal analysis, Data curation. **Yuji Teramura:** Writing – review & editing, Visualization, Resources, Methodology, Investigation, Data curation, Conceptualization. **Shinichiro Shinzaki:** Writing – review & editing, Methodology, Investigation.

## Declaration of competing interest

The authors declare that they have no known competing financial interests or personal relationships that could have appeared to influence the work reported in this paper.

## Data Availability

Data will be made available on request.
