# Supplementary material for: Cell patterning in vivo using microrobot specifically designed for tissue engineering applications
Source: Mater Today Bio. 2025 Dec 17;36:102683. doi: 10.1016/j.mtbio.2025.102683 (PMC12813073; doi:10.1016/j.mtbio.2025.102683)
Supplement: Multimedia component 1 [file mmc1.docx]

**Supporting Information**

**Cell patterning *in vivo* using microrobot specifically designed for tissue engineering applications**

Hironori Yamazoe^1^*, Yoshiaki Yamano^1^, Yuji Teramura^2^, Shinichiro Shinzaki^3^

^1^ Molecular Biosystems Research Institute, National Institute of Advanced Industrial Science and Technology (AIST), 1-8-31 Midorigaoka, Ikeda, Osaka 563-8577, Japan

^2^ Cellular and Molecular Biotechnology Research Institute, National Institute of Advanced Industrial Science and Technology (AIST), 1-1-1 Higashi, Tsukuba, Ibaraki 305-8566, Japan

^3^ Department of Gastroenterology, Faculty of Medicine, Hyogo Medical University, 1-1 Mukogawa, Nishinomiya, Hyogo 663-8501, Japan

*Correspondence to: Hironori Yamazoe, National Institute of Advanced Industrial Science and Technology (AIST), 1-8-31 Midorigaoka, Ikeda, Osaka 563-8577, Japan

Email: hironori-yamazoe@aist.go.jp

Tel.: +81-50-3522-6825, Fax: +81-72-789-1454

**
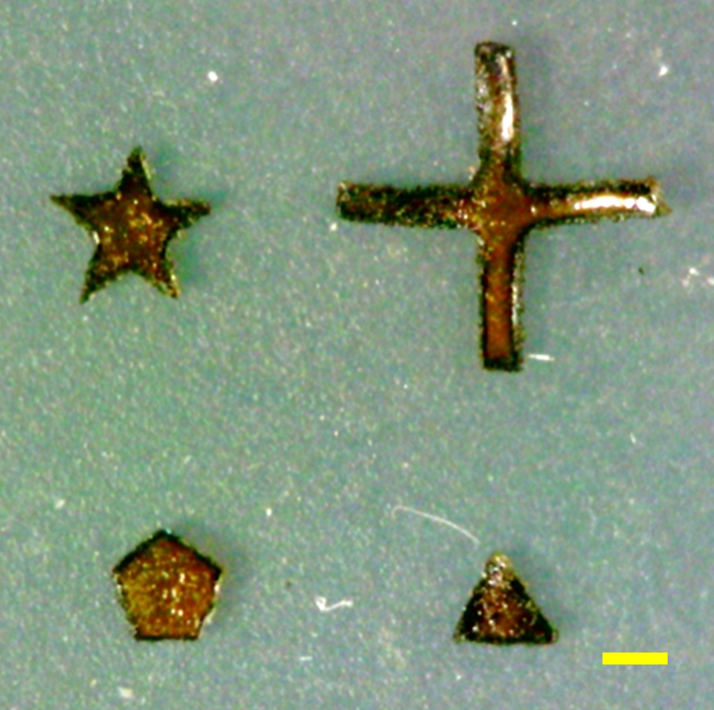
**

**Figure S1.** Images of robot bodies with various shapes. Each shape was prepared by cutting the albumin film using a laser cutter. Scale bar: 1 mm.

**Elemental characterization of microrobot body**

Figure S2a shows the low-magnification energy-dispersive X-ray spectroscopy (EDS) mapping of the microrobot body, including a portion of the sample stage for comparison. The iron signal (represented by the red dots in the map) was uniformly distributed across the microrobot structure, thus confirming the even incorporation of the maghemite (γ-Fe_2_O_3_) magnetic nanoparticles (MNPs). By contrast, no iron signal was detected in the area corresponding to the sample stage, thereby verifying the origin of the iron content within the microrobot.

Separately, the quantitative EDS analysis performed at a high magnification revealed the elemental composition of the microrobot body, which was determined to be C: 27.2%, N: 5.7%, O: 37.8%, S: 0.7%, and Fe: 28.6% (weight %) (Figure S2b).


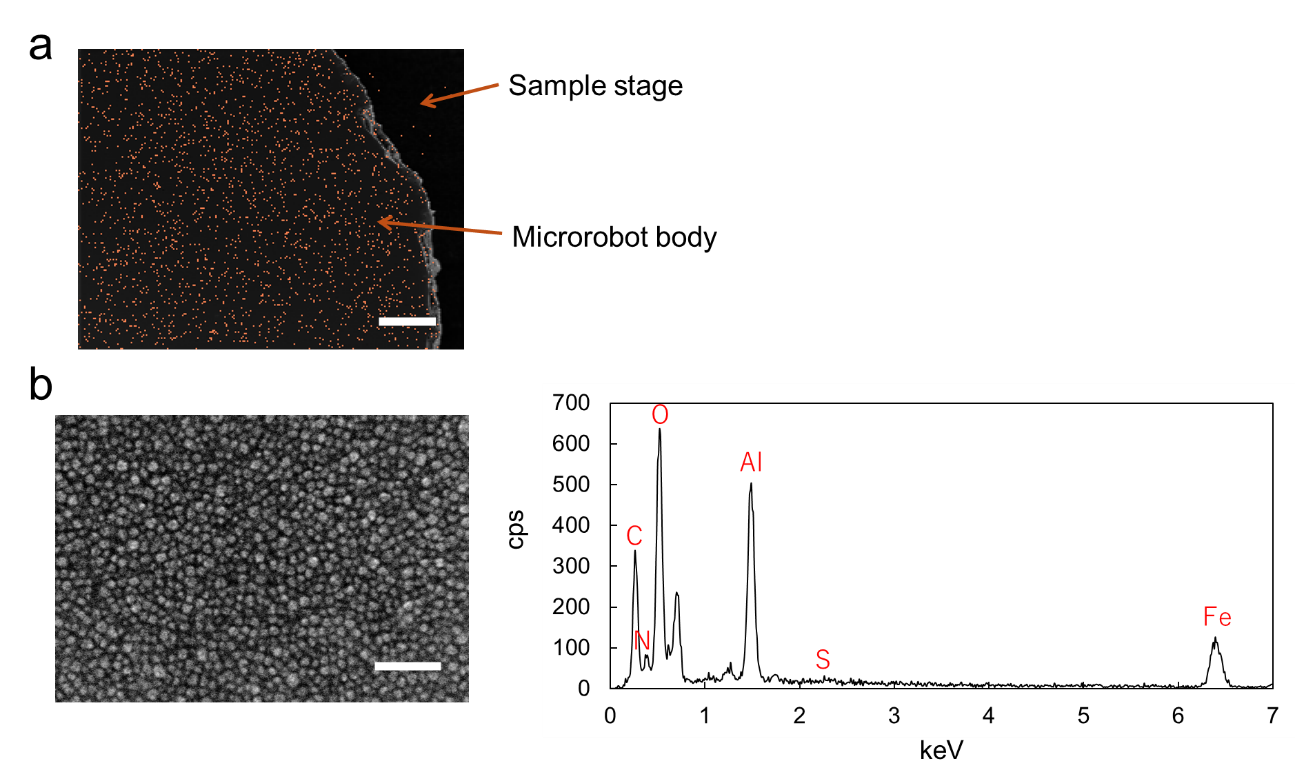


**Figure S2.** (a) EDS elemental mapping of the microrobot body. Iron is represented as red dots. Scale bar: 2 μm. (b) EDS spectrum obtained from the microrobot body, along with the corresponding scanning electron microscopy (SEM) image. Scale bar: 200 nm. Notably, although an aluminum peak was observed in the EDS spectrum, it originated from the sample stage and was not an intrinsic component of the microrobot.

**Examination of th****e surface coverage of the cell membrane-anchoring reagent (CMAR) using a fluorescent labeling dye**

The CMAR was covalently immobilized on the surface of the microrobot body *via* its amine-reactive groups. The robot body was treated with a green amine-reactive fluorescent labeling dye to examine the presence of the corresponding reaction sites on the microrobot surface. As shown in Figure S3a, clear green fluorescence was observed across the entire microrobot surface, confirming the abundance of reaction sites. Conversely, no clear fluorescence signal was observed in the microrobot after CMAR immobilization (Figure S3b). This result indicated that CMAR was successfully immobilized across the entire surface, consuming the available reaction sites.

**
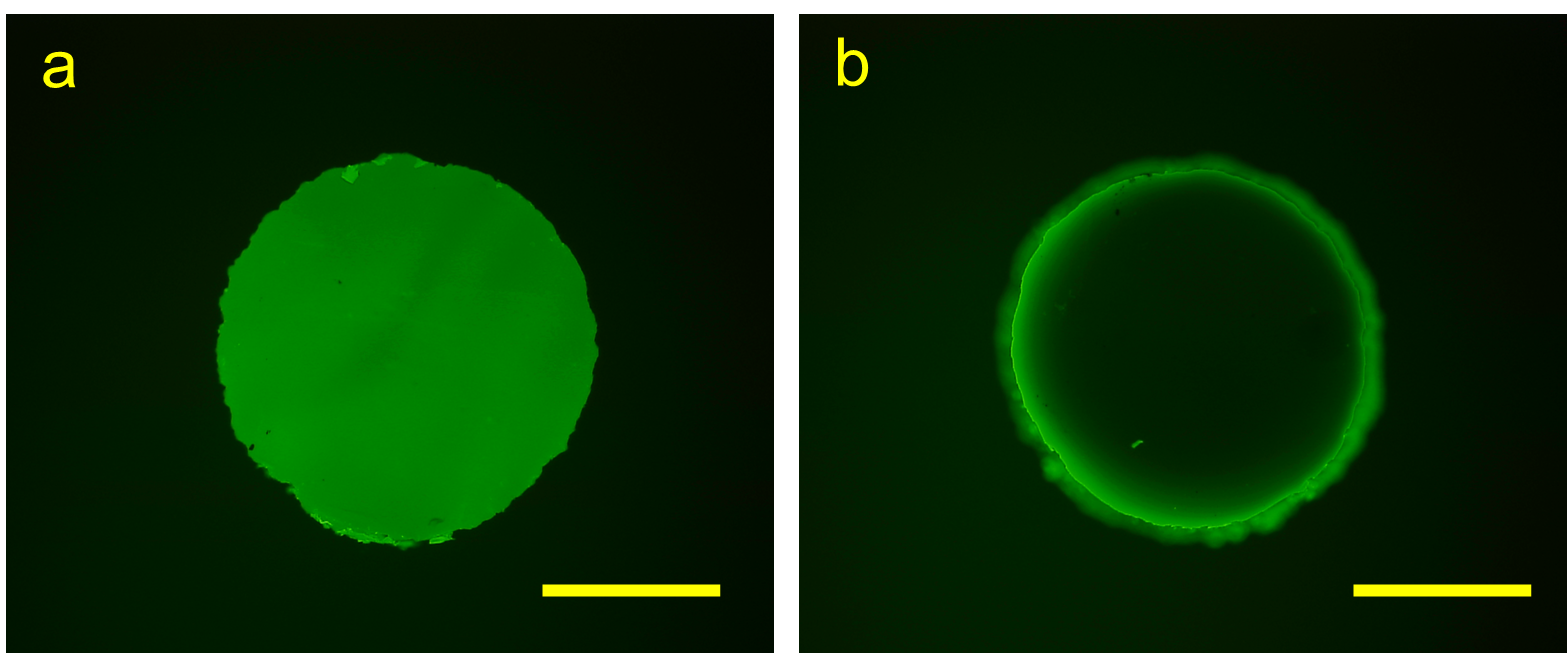
**

**Figure S3.** Fluorescence images of the microrobot stained with an amine-reactive green fluorescent dye: (a) before and (b) after CMAR immobilization. Scale bar: 300 μm. Notably, the faint fluorescence signal observed at the edges of the microrobot in Figure S3b was thought to be an optical artifact.

**Immobilization of CMAR onto surface of robot body**

The amine-reactive group in CMAR reacts with the ε-amines of the lysine side chains and α-amines at the N-terminus of albumin molecules on the surface of robot body. We examined the binding of the CMAR to robot bodies using quartz crystal microbalance (QCM) measurements. The frequency dynamics as a function of time in response to addition of 0.5 or 1 μM CMAR are shown in Figure S4. A large decrease in frequency was observed for both samples, confirming the immobilization of CMAR on the surface of the robot bodies. The amount of immobilized CMAR 30 min after addition of 0.5 and 1 μM CMAR was estimated to be 46 ± 3 ng/cm^2^ (corresponding to 0.7 × 10^13^ CMAR molecules/cm^2^) and 83 ± 6 ng/cm^2^ (corresponding to 1.3 × 10^13^ CMAR molecules/cm^2^), respectively, using Sauerbrey equation. Thus, the amount of CMAR in the robot for NIH3T3 cells prepared using 1 μM CMAR was 1.8-fold higher than that for mesenchymal stem cells (MSCs) prepared using 0.5 μM CMAR.


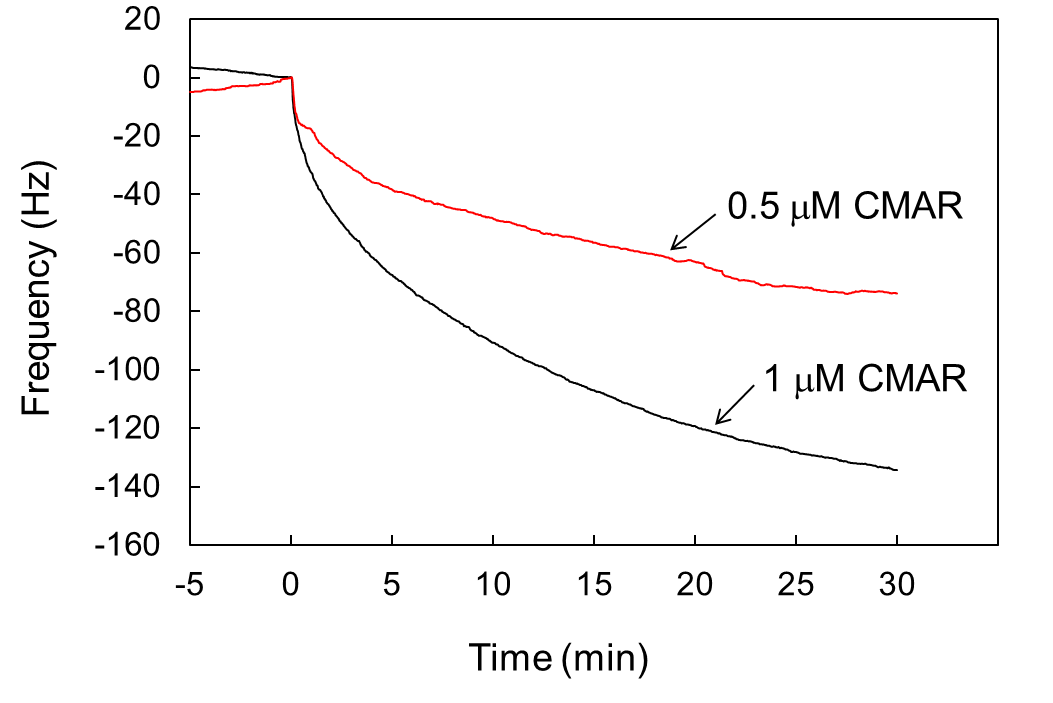


**Figure S4.** Typical time courses of frequency changes after addition of 0.5 or 1 μM CMAR.

**Area of the single cell captured by the microrobot**

Figure S5 shows a magnified view of the NIH3T3 cells and MSCs captured by the microrobot. The area of a single cell was 142 ± 44 μm^2^ for NIH3T3 cells and 309 ± 52 μm^2^ for MSCs. Thus, the area of MSCs was 2.2-fold larger than that of NIH3T3 cells.

As described in Figure S4, 1.3 × 10^13^ and 0.7 × 10^13^ CMAR molecules/cm^2^ were present on the microrobot for NIH3T3 cells and MSCs, respectively. As a result, single NIH3T3 cells with an area of 142 μm^2^ and MSCs with an area of 309 μm^2^ are expected to interact with a similar number of CMAR molecules (1.8 × 10^7^ and 2.2 × 10^7^ CMAR molecules for NIH3T3 cells and MSCs, respectively).


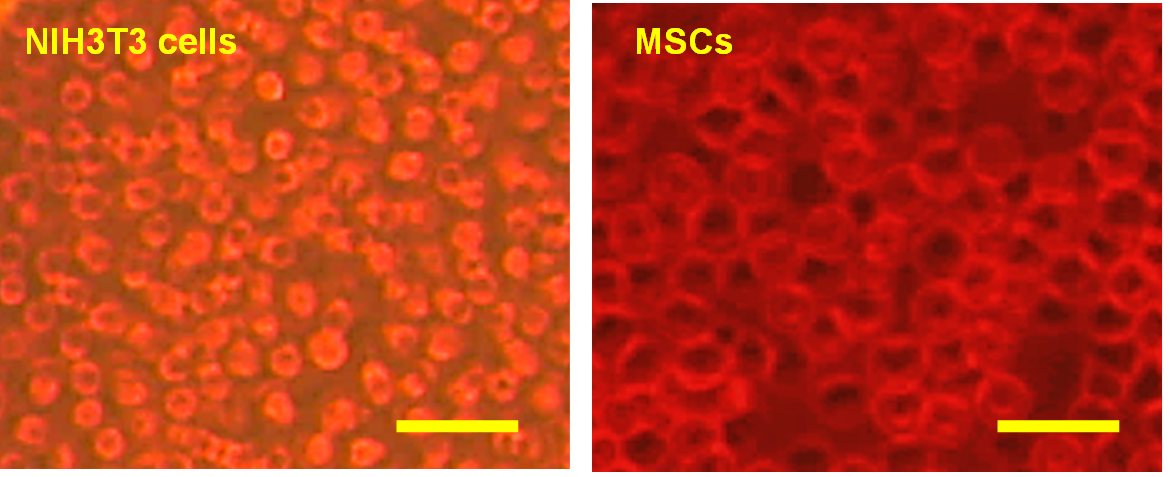


**Figure S5.** Magnified views of NIH3T3 cells and MSCs captured by the microrobot. Scale bar: 50 μm.


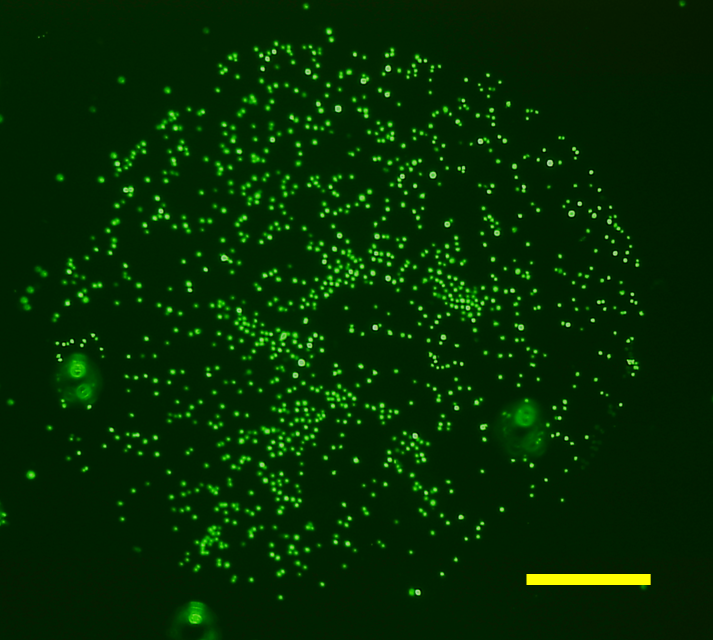


**Figure S6.** Representative fluorescence image of the circular pattern of NIH3T3 cells created on the collagen gel using the circular robot with a 2 mm diameter. The percentage of cell coverage of NIH3T3 cells within the predetermined circular pattern was 25 ± 7 %. Scale bar: 500 μm.

**Modification of cell surface property**

Teramura et al. synthesized CMARs comprising three different units: peptides with various sequences, a polyethylene glycol spacer, and hydrophobic lipids (Figure S7a) [1]. The lipids in CMAR are incorporated into the cell membrane through hydrophobic interactions, allowing for immobilization of various peptides on the cell surface. A previous study showed that the attachment of floating cells to a polystyrene culture dish with a negatively charged surface was promoted by immobilizing positively charged peptides but not neutral peptides [1]. Based on these results, positively charged peptides were immobilized on the surface of cells captured by the robot (Figure S7b). Using the surface-modified cells, precise cellular patterns were created on a polystyrene culture dish (Figure S7c). Diverse molecules such as peptides, proteins, and DNA can be immobilized on the cell surface using this strategy [1-3]. Therefore, if required, the precision of cellular patterns can be improved by modifying the cell surface properties in accordance with the physicochemical properties of the target surfaces.

**
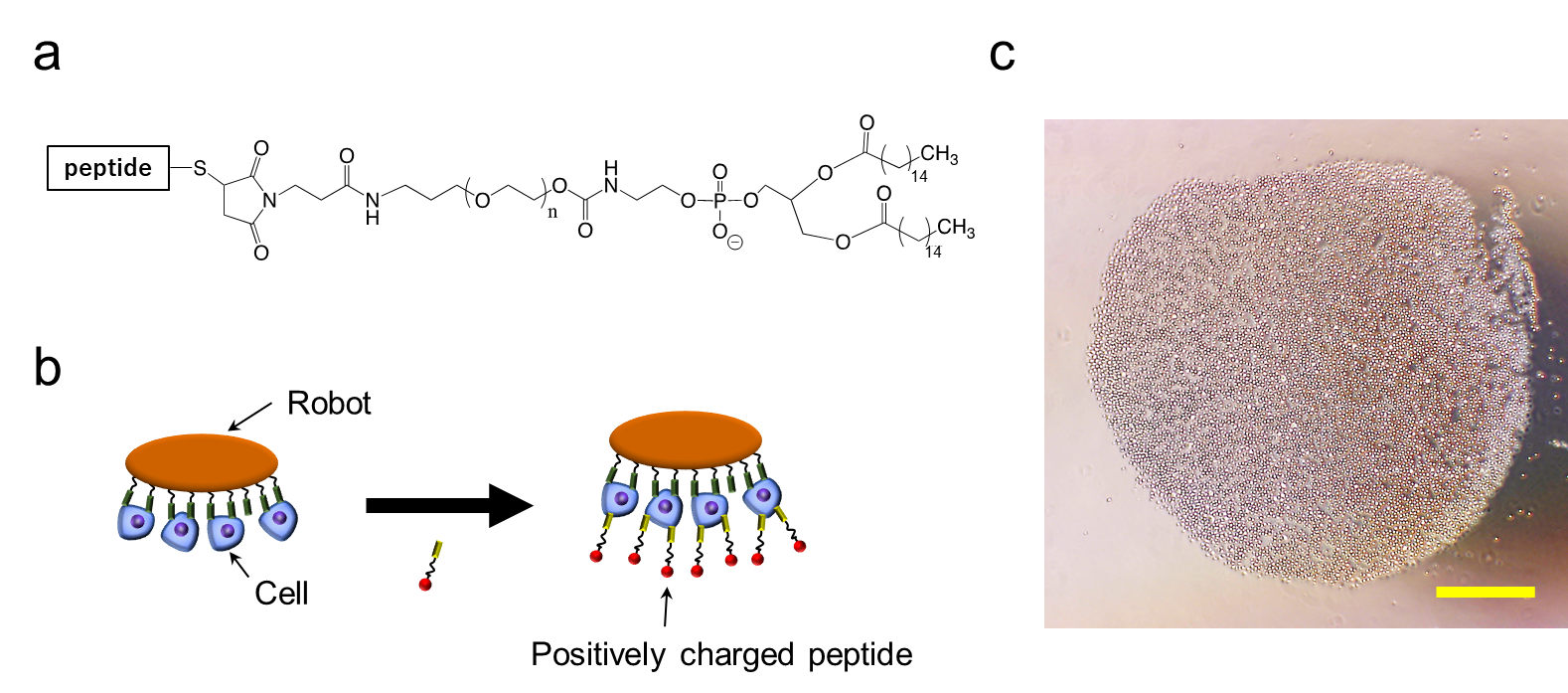
**

**Figure S7.** (a) Chemical structure of synthesized CMAR. (b) To immobilize the positively charged peptide to the cell surface, an NIH3T3 cell-loaded circular robot with a 2 mm diameter was immersed in 500 μg/mL solution of CMAR containing a positively charged peptide with the sequence YGRKKRRQRRRC, 40 kDa polyethylene glycol spacer, and lipids in PBS for 15 min. (c) Phase-contrast image of the circular pattern of NIH3T3 cells created on the culture dish. Scale bar: 500 μm.

**Microrobot resistance to enzymatic degradation**

Trypsin was used as a model protease to test the enzymatic degradability of the microrobots. Figure S8 shows that the microrobots exhibited no significant morphological changes after 24 hours of incubation with a high concentration of trypsin solution at 37 °C, thus indicating the high stability of microrobot against enzymatic digestion. This structural integrity is attributable to the robust covalent bonds formed between albumin molecules. In addition, CMAR immobilized on the microrobot surface may prevent trypsin from accessing the albumin-based robot body.

We used amine-functionalized MNPs, which react with epoxy cross-linkers, thereby enabling the formation of covalent bonds between albumin and MNPs. Although the detailed molecular mechanism of this interaction is yet to be fully elucidated, we hypothesize that the cross-linkers that react with albumin *via* one epoxy group, while retaining another intact epoxy group, persist in the cross-linked albumin solution. The remaining intact epoxy groups subsequently facilitated the formation of covalent bonds between albumin and the MNPs when the crosslinked albumin solution and MNPs were mixed.

**
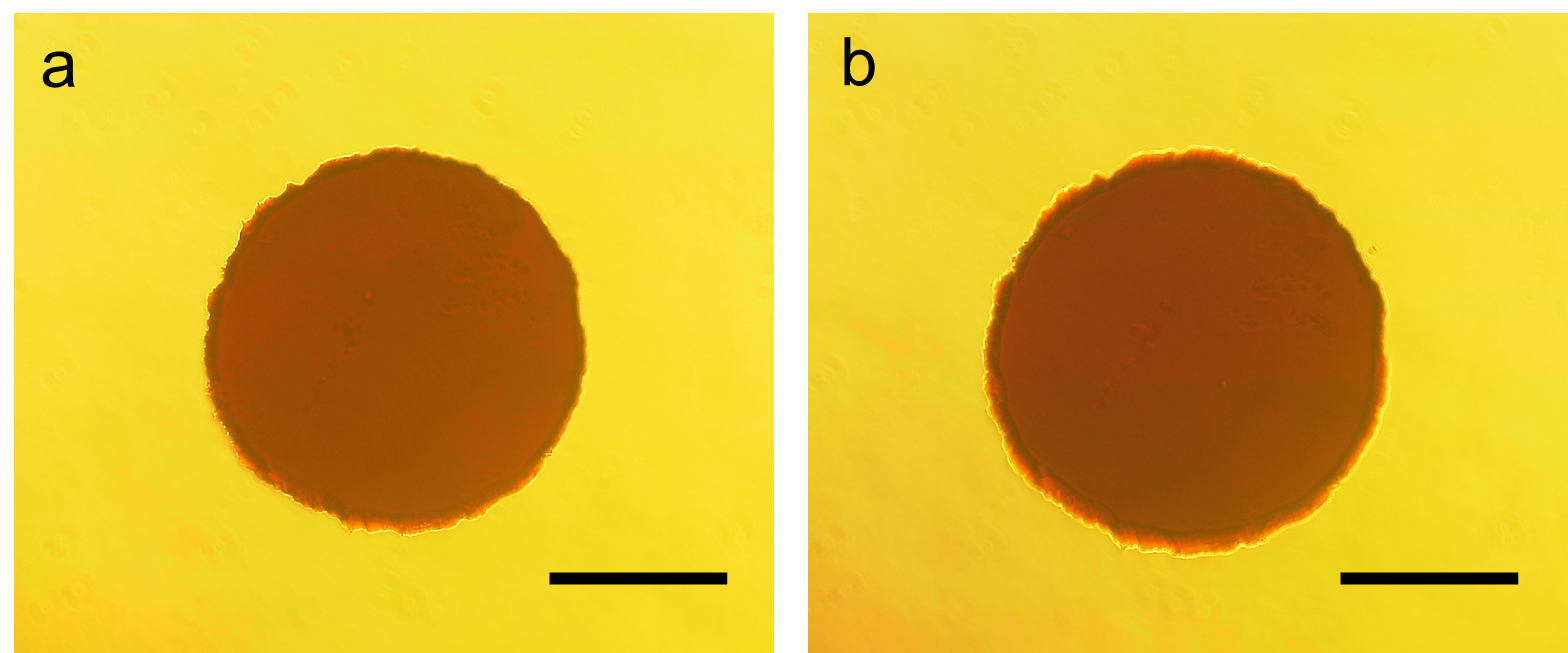
**

**Figure S8.** Phase-contrast micrographs of the microrobots (a) before and (b) after incubating with 1 mg/mL trypsin solution for 24 h at 37 °C. Scale bar: 300 μm.

**
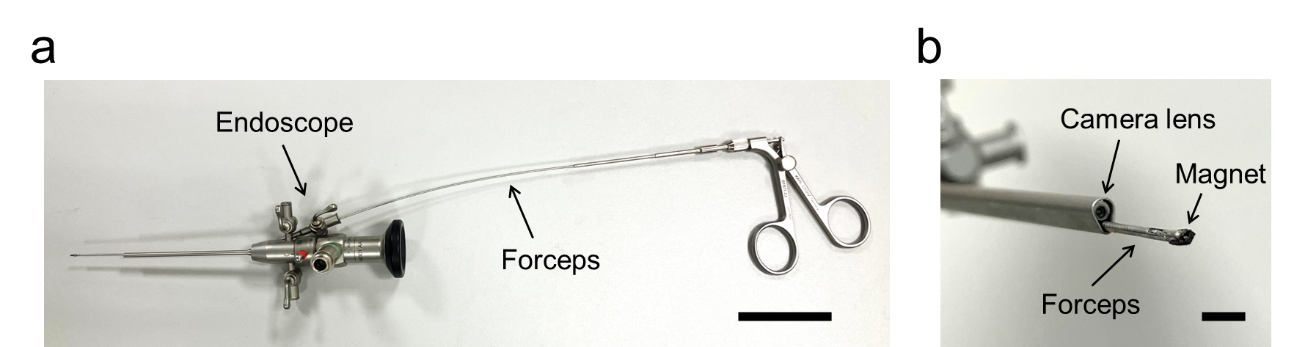
**

**Figure S9.** (a) Photographs of an endoscope and the inserted magnet-attached forceps. Scale bar: 5 cm. (b) Magnified view of the tip of endoscope. Scale bar: 5 mm.

***In vitro* physical stress assay**

In the colon, patterned cells are thought to be exposed to various physical stimuli such as the passage of feces and other solid matter. In order to simulate this physical stress *in vitro*, we added small beads to MSC-cultured plates and laterally shaken them for 1 h. This stimulation resulted in the death of approximately 32% of exposed cells (Figure S10). In contrast, when the cells were covered with collagen gel, they were protected from shaking-bead-induced damage and exhibited high viability.


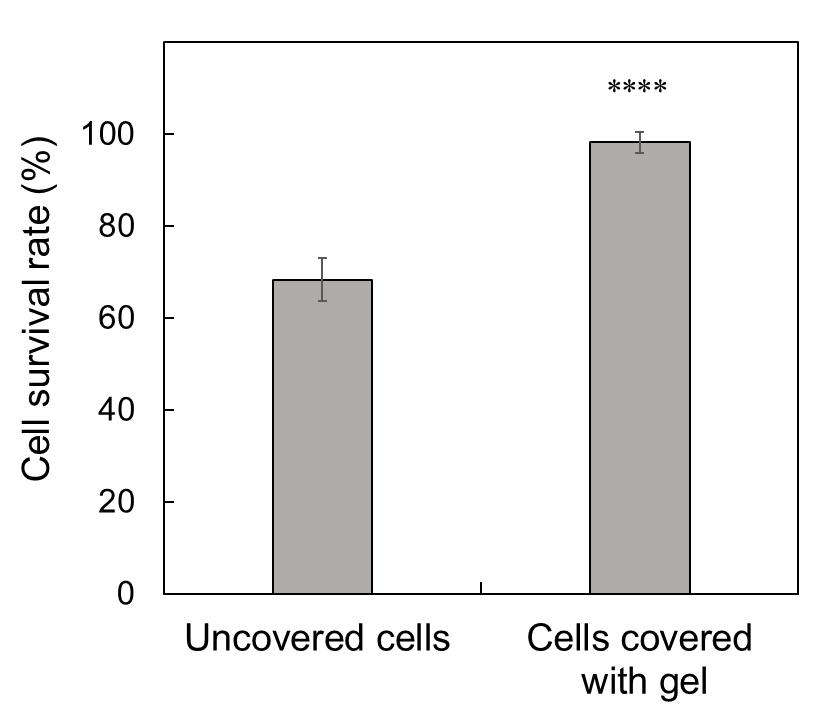


**Figure S10.** Cell survival rate of MSCs with or without collagen gel covering after exposure to physical stress. Data are shown as the means ± SD (n = 6). **** *p* < 0.0001 vs. uncovered cells by two-sided Welch's *t*-test.

**Movie S1:** Magnetic attraction of a circular microrobot with 500 μm diameter in water using a neodymium magnet (diameter, 10 mm; height, 20 mm; magnetic induction, 566 mT). Scale bar: 5 mm.

**Movie S2:** Endoscopic observation of the colon of the healthy control mice. Movies were recorded while the endoscope was withdrawn slowly.

**Movie S3:** Endoscopic observation inside the colon of dextran sulfate sodium (DSS)-treated mouse.

**Movie S4:** Release of the circular microrobot with a diameter of 500 μm from the endoscope. Scale bar: 5 mm.

**Movie S5:** Endoscopic observation of MSC-loaded circular microrobot 500 μm in diameter placed inside the colon of DSS-treated mouse.

**Movie S6:** Retrieval of a circular microrobot with a diameter of 500 μm using endoscopic forceps with a magnet attached to its tip. Scale bar: 3 mm.

**References**

[1] Y. Teramura, S. Asif, K. N Ekdahl, E. Gustafson, B. Nilsson, Cell adhesion induced using surface modification with cell-penetrating peptide-conjugated poly(ethylene glycol)-lipid: A new cell glue for 3D cell-based structures. ACS Appl. Mater. Interfaces 9 (2017) 244-254.

[2] K. Sakurai, Y. Teramura, H. Iwata, Cell immobilized on patterns printed in DNA by an inkjet printer. Biomaterials 32 (2011) 3596-3602.

[3] Y. Teramura, H. Iwata, Islet encapsulation with living cells for improvement of biocompatibility. Biomaterials 30 (2009) 2270-2275.
